# Acetone Sensing Properties and Mechanism of SnO_2_ Thick-Films

**DOI:** 10.3390/s18103425

**Published:** 2018-10-12

**Authors:** Yanping Chen, Hongwei Qin, Yue Cao, Heng Zhang, Jifan Hu

**Affiliations:** 1 School of Physics, State Key Laboratory for Crystal Materials, Shandong University, Jinan 250100, China; yanping_c@sdjzu.edu.cn (Y.C.); 201611428@mail.sdu.edu.cn (Y.C.); 201411433@mail.sdu.edu.cn (H.Z.); 2 School of Science, Shandong Jianzhu University, Jinan 250101, China

**Keywords:** gas sensors, acetone, SnO_2_, sensing mechanism

## Abstract

In the present work, we investigated the acetone sensing characteristics and mechanism of SnO_2_ thick-films through experiments and DFT calculations. SnO_2_ thick film annealed at 600 °C could sensitively detect acetone vapors. At the optimum operating temperature of 180 °C, the responses of the SnO_2_ sensor were 3.33, 3.94, 5.04, and 7.27 for 1, 3, 5, and 10 ppm acetone, respectively. The DFT calculation results show that the acetone molecule can be adsorbed on the five-fold-coordinated Sn and oxygen vacancy (V_O_) sites with O-down, with electrons transferring from acetone to the SnO_2_ (110) surface. The acetone molecule acts as a donor in these modes, which can explain why the resistance of SnO_2_ or n-type metal oxides decreased after the acetone molecules were introduced into the system. Molecular dynamics calculations show that acetone does not convert to other products during the simulation.

## 1. Introduction

Over a period of three decades, semiconductor metal oxide gas sensors have been extensively investigated due to their stable chemical transduction properties, which can reversibly convert chemical interactions on a surface to change the conductivity. Surface conduction is regulated by the adsorption and desorption of gas molecules on the surface of materials. Among the semiconductor metal oxides used in gas sensors, SnO_2_ has received considerable attention in science and technology for many years. SnO_2_ with a wide band gap of 3.6 eV is a significant functional material applicable for solar cells, catalysis, transparent electrodes, and, particularly, in gas sensor devices because of its unique optical, catalytic, and electrical properties [1,2,3,4,5,6]. It has been widely used to detect toxic chemicals such as CH_4_, H_2_, C_2_H_5_OH, gasoline, CO, C_2_H_2_, NO_2_, NO, and H_2_S [7]. As the most promising sensor, it is highly desirable to improve the sensing properties of SnO_2_ and meet the needs of sensors to work in more complicated environments. The sensing ability of the sensor to specific gas depends on the interaction between the gas molecules and the sensor surface. The reactivity of the surface is critically dependent on the doping or defect structures. Notably, it has been proven that proper doping is one of the most effective ways to enhance the gas sensing properties by modifying the geometric and electronic structures of the surface.

Oxygen-related gas sensing generally involves the chemisorption of oxygen on the surface of SnO_2_, which then undergoes charge transfer during the reaction of chemisorbed oxygen with the target gas molecules, thereby changing the surface resistance of the sensor element [8]. The electrons trapped by oxygen deplete the surface of the charge carrier and form a surface potential to act as a potential barrier against electron flow. Inside the sensor, current flows through the conjunction parts (grain boundary) of SnO_2_ microcrystals. The adsorbed oxygen forms a potential barrier at the grain boundary that prevents the free movement of carriers, and the increase in resistance of the SnO_2_ film is attributed to this potential barrier. If a reducing gas is present in the air sample, it reacts with the adsorbed oxygen species. In this way, free electrons are released back to the conduction band, and the surface density of negatively charged oxygen decreases. As a result, both the grain boundary barrier height and the sensor resistance are reduced [9,10,11,12,13].

Acetone is a commonly used reagent in industry and laboratories. It is highly unstable and is greatly harmful to human health and biology. Inhalation of acetone can cause headaches, allergies, fatigue, and even narcosis [14,15,16,17], and can be harmful to the nervous system. Therefore, for the sake of safety, it is urgent to monitor the concentration of acetone in the environment and workplace. In addition, the use of exhaled gas from humans to diagnose diabetes has great promise as it is non-invasive [18]. Acetone can be used as a breath marker for the diagnosis of diabetes because the concentration of acetone in healthy individuals’ breath varies from 0.3 to 0.9 ppm and in the exhaled air of diabetic patients exceeds 1.8 ppm [18]. A chemiresistive type sensor using SnO_2_ is considered as an exhalation gas sensor because of its excellent reactivity with volatile organic compounds (VOCs), easy fabrication processes, and the possibility of miniaturization of portable integration [3,4,19,20,21,22,23,24,25,26]. For example, L. Cheng et al. [3] have developed Y-doped SnO_2_ hollow nanofibers for the detection of acetone. L.P. Qin et al. [4] reported the effect of temperature on the acetone-sensing properties of SnO_2_ nanowires. Also, J. Zhao et al. [19] reported the acetone vapor sensing performance of SnO_2_ thin films prepared by dip-coating. In addition, as reported by S.B. Patil et al. [20], the addition of cobalt to the SnO_2_ thin films can help increase the active surface area and thereby increase the absorption of test gases. S. Singkammo et al. [21] also developed Ni-doped SnO_2_ sensors for the detection of acetone, in which the SnO_2_ nanoparticles were prepared by spin-coating. The understanding of gas-surface interactions at the atomic level and the study of SnO_2_ semiconductor gas sensor mechanisms have attracted more and more attention [27,28,29,30,31,32,33]. Among them, density functional methods have been successfully used to study surface geometry [27,28,29,30,31], the electronic and chemical properties of bulk and surface systems [32], and the reaction processes of adsorbents, such as H_2_ [30], O_2_ [33], NO_x_ [34], and C_2_H_5_OH [35] on a stoichiometric or oxygen-deficient SnO_2_ (110) surface.

In this paper, the acetone sensing characteristics and mechanism of SnO_2_ thick film are reported from experiments and first-principles methods. The microstructure and morphology of the as-synthesized SnO_2_ particles were analyzed by XRD and TEM. The sensing mechanism of the above SnO_2_ thick film is also discussed.

## 2. Materials and Methods

The nanocrystalline SnO_2_ powders were prepared by a co-precipitation method. SnCl_4_∙5H_2_O (99%, obtained from Beijing 57601 Chemical (Beijing, China)) was dissolved in deionized water. An appropriate amount of NH_3_ (25–28%, obtained from Kangde Chemical (Laiyang, China)) was added dropwise to the vigorously stirred solution. All materials were used without further purification. The final pH value was adjusted to approximately eight to promote a complete precipitation. The resulting slurry was filtered, washed with distilled water repeatedly, and finally dried in air at 120 °C for 20 h. The dried powders were milled and preheated at 350 °C for 4 h. Then, the powders obtained above were annealed in an oven at 400, 500, 600, 700, and 800 °C for 5 h to obtain the SnO_2_ nanoparticles. The structures of the resultant powders were characterized by means of X-ray powder diffraction (XRD, Model: Smart APEX II X-ray diffractometer with CuK_α_ source: λ = 0.15418 nm) and transmission electron microscopy (TEM, Model: Tecnai G2 F20).

**Figure 1:**
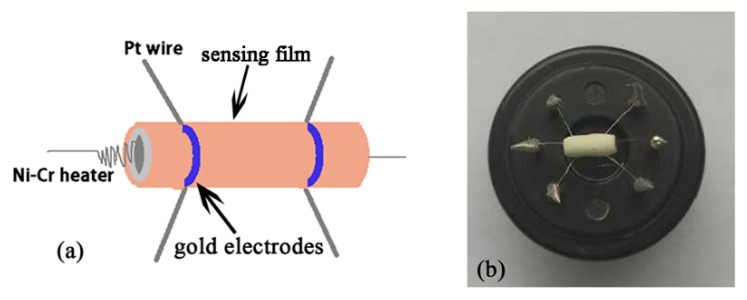
(**a**) The schematic diagram of a typical gas sensor; (**b**) the photo of the prepared sensor.

The powders were mixed with deionized water to form a paste. The obtained paste was then coated onto a prefabricated Al_2_O_3_ tube to form a thick film and air-dried for 1 h. The length and diameter of the tube were 4 mm and 1.2 mm, respectively. Two Au electrodes were installed at each end of the ceramic tube. Electric contacts were made with two platinum wires attached to the electrodes. A Ni-Cr heating wire was placed through the tube to control the operating temperature. The sensors were annealed at 240 °C for 48 h on the aging equipment in ambient air. The schematic diagram of a typical gas sensor and a photo of the prepared sensor are shown in Figure 1, respectively. The gas sensing tests were carried out on a computer-controlled WS-30A system (Wei Sheng Electronics Co. Ltd., Zhengzhou, China), and the measurement (upgraded and modified according to our experimental needs) was processed by a static process. The sensors were put into a glass chamber (20 L) at the beginning. When the resistances of all the sensors were stable (relative humidity was approximately 27%), the calculated amount of the acetone liquid was injected into the glass chamber by a micro-injector and mixed with air. After the sensor resistances reached a new constant value, the test chamber was opened to recover the sensors in air. The temperature of the sensor was also controlled by a computer. The sensor resistance and response values were acquired by the analysis system automatically. The various environmental relative humidities (RH) for the sensor response measurement in a closed test chamber were achieved at room temperature. Three elements were prepared for each sample, and the average gas sensitivity of the three elements was taken as the gas sensitivity of the sample. The gas-sensing response *S* in the experiment was defined as *R_a_/R_g_*, where *R_a_* and *R_g_* were the electric resistance in air and test gas, respectively.

## 3. Results and Discussion

**Figure 2:**
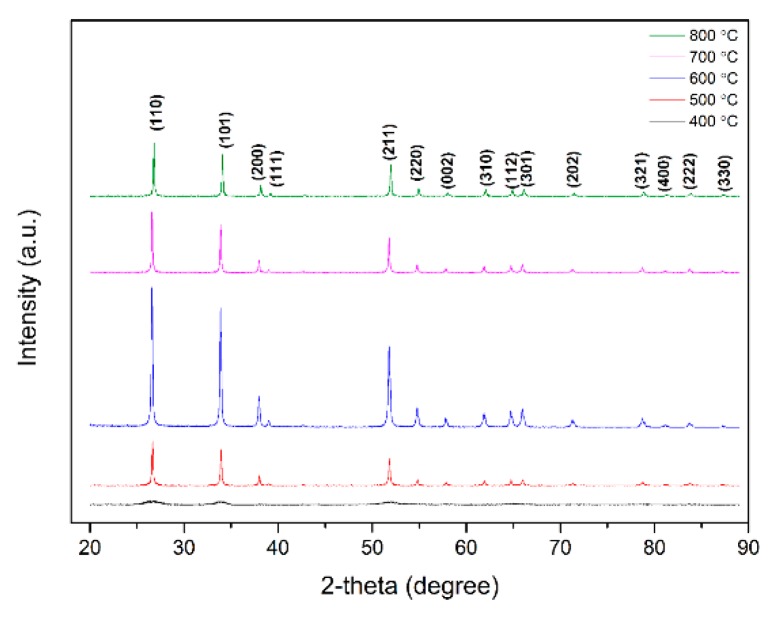
The X-ray diffraction pattern of the as-obtained SnO_2_ annealed at different temperatures.

The X-ray diffraction patterns of the powders annealed at 400, 500, 600, 700, and 800 °C for 5 h are shown in Figure 2. As can be seen from the figure, the tin oxide annealed at 400 °C is amorphous. The powders annealed at other temperatures crystallized as one single phase with the tetragonal SnO_2_ rutile structure with lattice constants of *a* = 4.738 Å and *c* = 3.188 Å (JCPDS file No: 21–1250) [36]. The average grain size *D* was estimated by means of the Scherrer method. The Scherrer equation is as follows:

(1) $$D=\frac{K\lambda }{\beta \cos\theta }$$

where *λ* is the wavelength of the incoming X-ray, *β* is the integral width of diffraction peaks, and *θ* is the Bragg diffraction Angle. When *β* is equal to half the width of the diffraction peak, *k* = 1 [37]. The obtained *D* values were about 19 nm, 10.1 nm, 11 nm, 17.6 nm, and 12.8 nm for SnO_2_ annealed at different temperatures.

**Figure 3:**
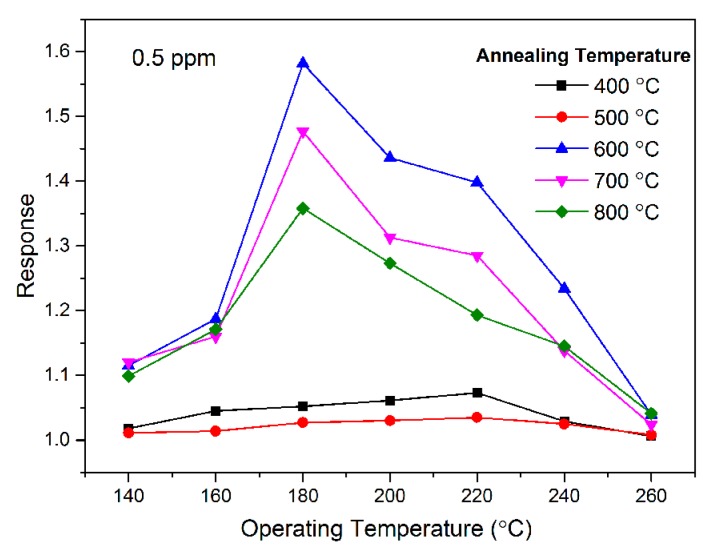
The response characteristics of the SnO_2_ thick-films as a function of the operating temperature.

It is well-known that the response of resistive sensors is considerably affected by the operating temperature, and it is obtained by performing the gas sensing experiments at different temperatures. The response characteristic of the SnO_2_ thick-films as a function of the operating temperature in the range 140–260 °C at 0.5 ppm of acetone vapor in air is shown in Figure 3. It can be seen that SnO_2_ may potentially be used as a sensing material for the detection of acetone at low concentrations. The responses of all SnO_2_ samples initially increase and attain the highest value and then decline with the operating temperature. The amount of gas adsorbed reaches an equilibrium at the appropriate temperature. Above this temperature, this equilibrium is interrupted, and the gas response is reduced [38]. This behavior can be explained from the kinetics and mechanics of gas adsorption and desorption on the surface of SnO_2_ [39]. The quantity of the chemically adsorbed gas species on the surface gradually increases with an increase in the operating temperature until the rate of desorption becomes equal to that of adsorption. The maximum amount of chemisorption is reached at the temperature with the highest gas response. If the temperature is further increased above this temperature, the balance will move to desorption, as the chemisorption is an exothermic reaction. Then the amount of adsorbed gas is reduced, resulting in a decreased gas response [40,41]. The response of SnO_2_ samples annealed at 400 °C and 500 °C attained a maximum value at 220 °C and the corresponding responses were 1.073 and 1.035, while SnO_2_ samples annealed at 600 °C, 700 °C, and 800 °C attained the maximum value at 180 °C and the corresponding responses are 1.582, 1.477, and 1.358, respectively. Therefore, 600 °C is the optimum annealing temperature and 180 °C is the optimum operating temperature. The data are summarized in Table 1. The optimum operating temperature of SnO_2_ shifts to a lower optimal working temperature when annealed at 600 °C, 700 °C, and 800 °C. The reduction of the optimal operating temperature of SnO_2_ may be caused by the large specific surface area, which increases the concentration of chemisorbed oxygen. Compared to others annealed at a higher temperature, the SnO_2_ sample annealed at 600 °C exhibits the highest acetone response. This may be ascribed to the smaller particle size of the SnO_2_ sample annealed at 600 °C. At 180 °C, the responses are 1.582, 1.477, and 1.273 for SnO_2_ sensors annealed at 600 °C, 700 °C, and 800 °C with 0.5 ppm acetone. Hereafter, we mainly conducted the gas sensing investigations of SnO_2_ sensors annealed at 600 °C.

**Figure 4:**
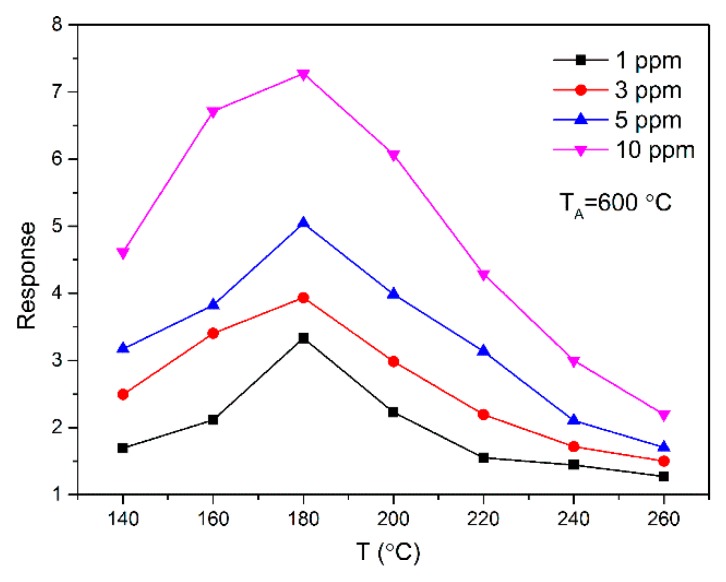
Response of sensors based on SnO_2_ nanoparticles versus operating temperature to 1, 3, 5, and 10 ppm acetone, respectively.

The response of the SnO_2_ sensor annealed at 600 °C to different concentrations of acetone in relation to the operating temperature is shown in Figure 4. T_A_ is the annealing temperature. We can see that the response increases continuously with an increase in the acetone concentration. This is attributable to the increased surface coverage of the acetone molecules on the membrane at higher concentrations, which promotes a subsequent reaction between acetone and atmospheric oxygen on the membrane surface, leading to a rapid chemical reaction and thus increasing the response. At the optimal operating temperature (T_O_) of 180 °C, the response of the SnO_2_ sensor was 3.333, 3.936, 5.043, and 7.274 for 1, 3, 5, and 10 ppm acetone gas, respectively.

**Figure 5:**
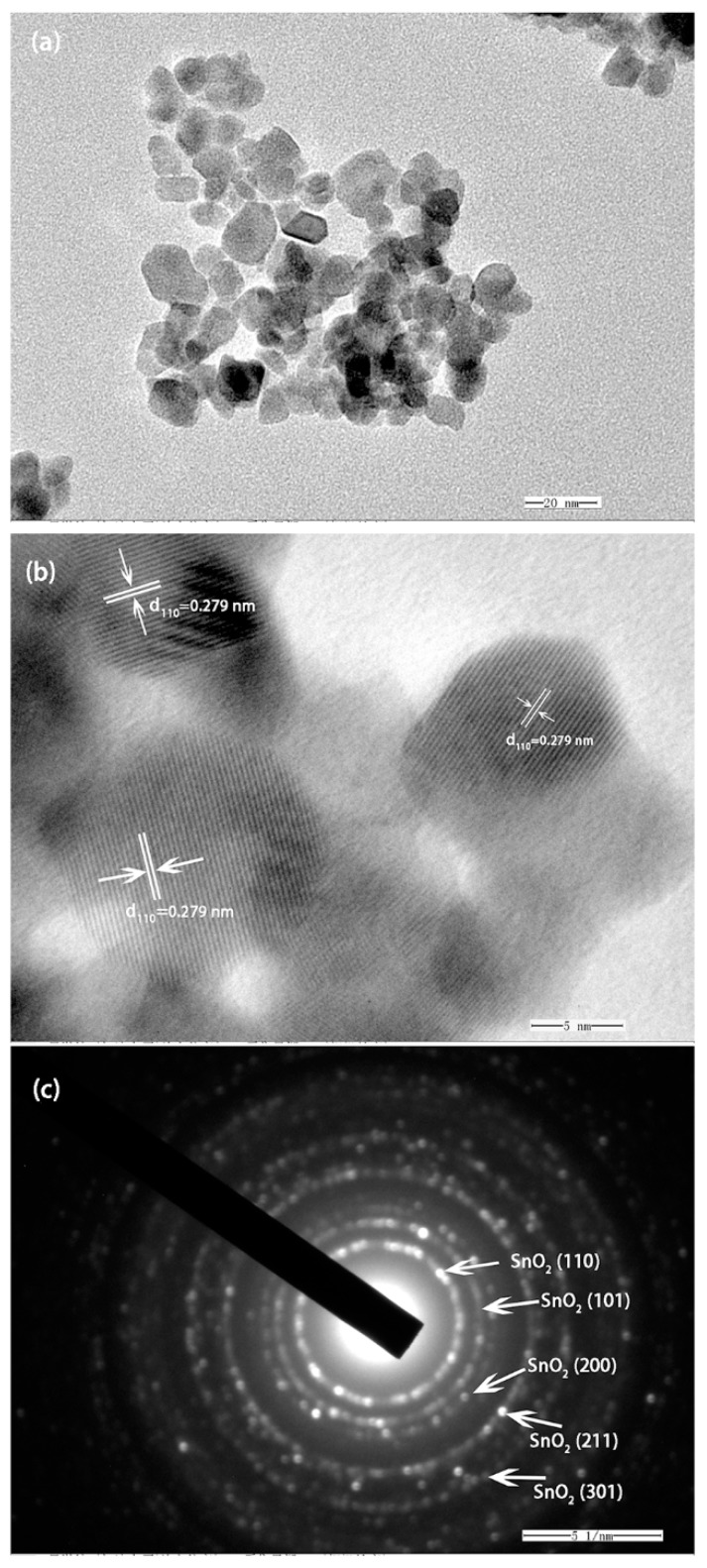
(**a**) TEM image of the SnO_2_; (**b**) HRTEM image of as-obtained SnO_2_ particles; (**c**) The selected area diffraction pattern of SnO_2_ particle.

Figure 5a–c shows the typical TEM observations of SnO_2_ nanoparticles annealed at 600 °C. A low-magnification TEM image, shown in Figure 5a, exhibited several typical SnO_2_ nanoparticles with diameters of 5.7 to 14.3 nm. A typical HRTEM image of a SnO_2_ nanoparticle is shown in Figure 5b, suggesting that the SnO_2_ nanoparticle was a single crystal. The interplanar distance value of ca. 0.279 nm can be readily assigned to the reflections from the (110) plane of tetragonal SnO_2_ [3,42]. The corresponding selected area diffraction (SAED) pattern, shown in Figure 5c, reveals a tetragonal SnO_2_ crystal with characteristic electron diffraction peaks of the (110), (101), (200), (211), and (301) planes, indicating high crystallinity of SnO_2_ nanoparticles in good agreement with the XRD data [9,42,43].

**Figure 6:**
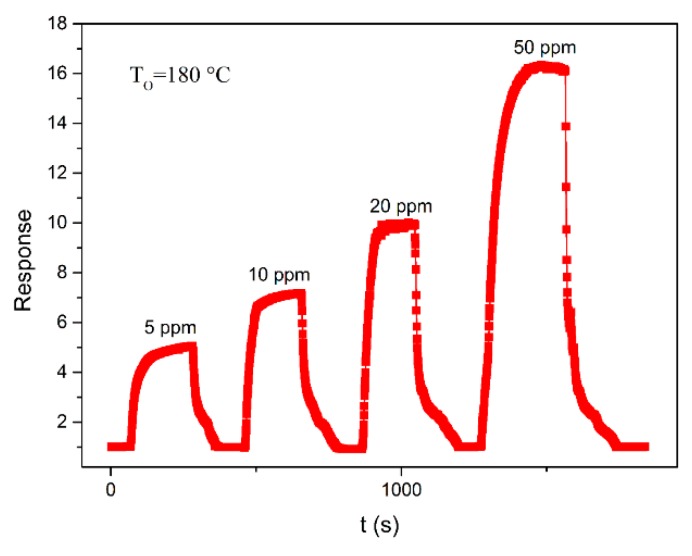
The dynamic sensing characteristics of the SnO_2_ sensor exposed to 5 ppm, 10 ppm, 20 ppm and 50 ppm acetone at an operating temperature of 180 °C.

Figure 6 shows the responses of the SnO_2_ sensor for 5, 10, 20, and 50 ppm acetone at the optimal operating temperature of 180 °C. It can be observed that the sensing response increases when the concentration of acetone increases from 5 to 50 ppm. After injection of 5 ppm, 10 ppm, 20 ppm, and 50 ppm acetone, the response of the sensor increased abruptly, corresponding to the responses of 5.043, 7.221, 10.6, and 16.898, respectively. The response then decreased rapidly and recovered to its initial value within a few minutes after the test gas was released. When injected with 5, 10, 20, and 50 ppm acetone, the response times of the sensor were 70, 38, 41, and 95 s, while the recovery times were 64, 90, 100, and 77 s, respectively. The response time and recovery time of SnO_2_ reported in the literature are summarized in Table 2. L. Cheng et al. [42] have reported that the response time and recovery time of three-dimensional hierarchical SnO_2_ nanoflowers for 500 ppm are 9 s and 7 s, respectively. V.V. Krivetsky et al. [43] have reported the response time and recovery time of the SnO_2_-based sensor for 1800 ppm acetone were 100 s and 500 s, respectively. Compared with the response-recovery time of SnO_2_ with the same morphology in the literature, the response time and recovery time of SnO_2_ in the prepared sample were reduced. For acetone vapor sensing, the oxygen species (O^−^, O_2_^−^, O^2−^) adsorbed on the SnO_2_ surface play an important role in the electrical transport properties. During the reaction process, acetone molecules donate electrons to the previously adsorbed oxygen species. Thus, the conductance of SnO_2_ will increase as acetone vapor is introduced into the test chamber due to the exchange of electrons between the oxygen species and SnO_2_ itself.

**Figure 7:**
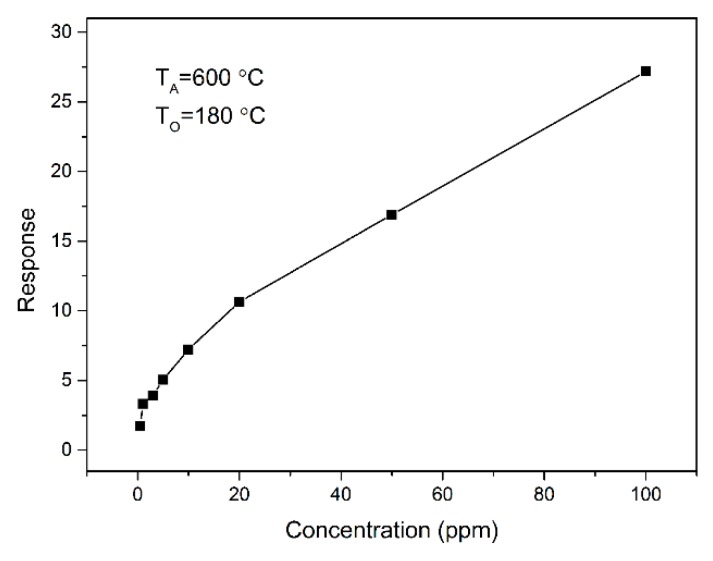
The response dependence on the acetone concentration for SnO_2_ powders at the operating temperature of 180 °C.

At the optimal operating temperature of 180 °C, the relationship between the sensitivity of the SnO_2_ thick film and the acetone vapor concentration is shown in Figure 7. With an increase in the acetone concentration, the response growth amplitude of the SnO_2_ sensor declines. The SnO_2_ sensor is more sensitive to low concentrations of acetone vapors which may be attributed to the availability of a sufficient number of sensing sites on the film to act. In comparison with the previous SnO_2_ reports [3,4,19,20,42,43,44,45,46,47,48,49,50], the corresponding properties for some other SnO_2_ sensors to acetone are shown in Table 2. After comparison, we can conclude that the SnO_2_ sensor we made can detect low concentrations with a high response at low temperature. Since the sensor is operated at elevated temperatures (180 °C), an additional circuit for temperature compensation should be included in the measurement setup.

**Figure 8:**
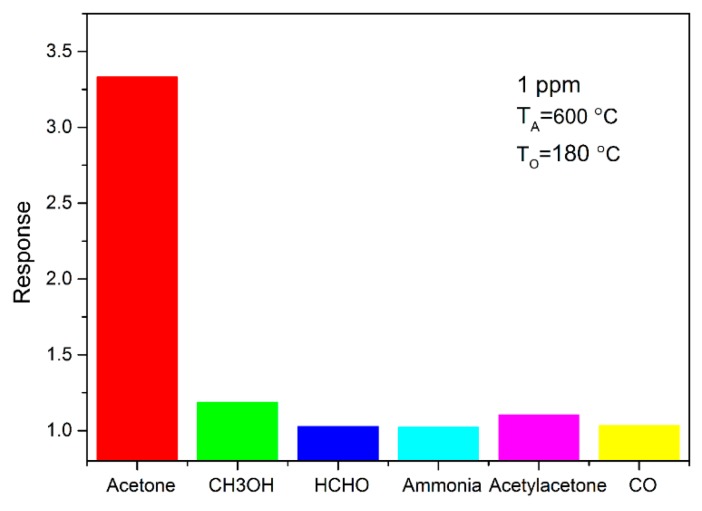
The response of SnO_2_ thick film to different gases with a concentration of 1 ppm at 180 °C.

**Figure 9:**
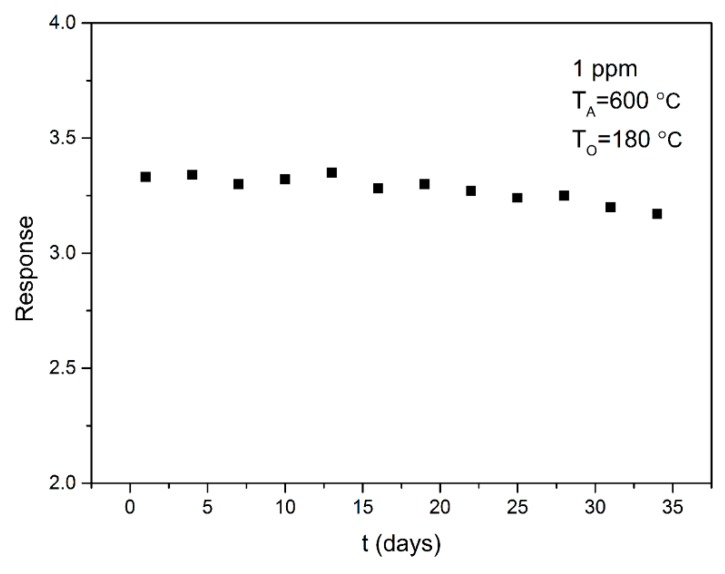
Response stability of SnO_2_ thick film sensor measured upon exposure to 1 ppm acetone gas for 33 days.

Selectivity is another important factor for gas sensors. The sensor selectivity was tested by exposing it to 1 ppm of different gases at 180 °C, and the test results are shown in Figure 8. It can be seen that the sensor shows a high response to acetone compared to other gases. The results indicate that the SnO_2_ based sensor shows good selectivity for acetone. Long-term stability is another important parameter for the gas sensor. The stability of the SnO_2_ sensor is measured at 1 ppm of acetone for 33 days, as shown in Figure 9. The result indicates that SnO_2_ annealed at 600 °C has good long-term stability, which benefits its practical application.

**Figure 10:**
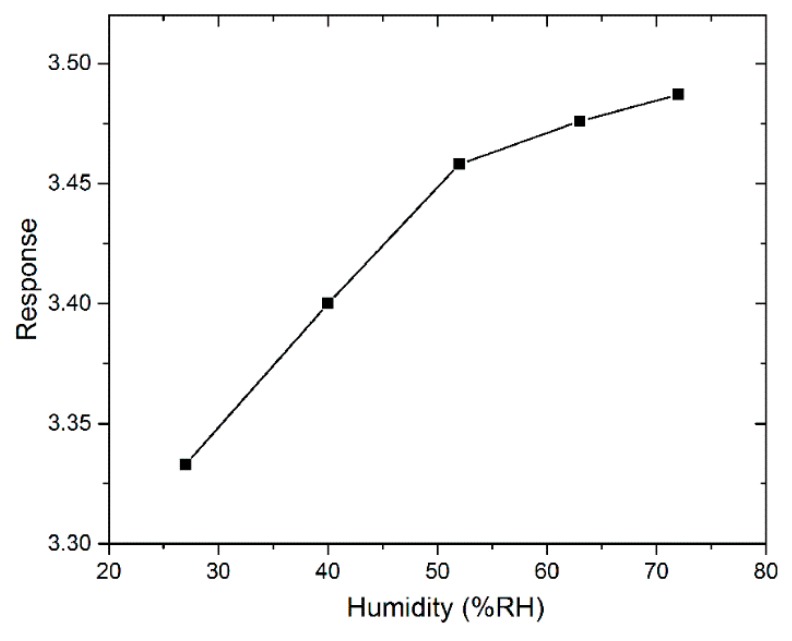
The humidity dependence of the sensing response to 1 ppm acetone at the operating temperature of 180 °C for SnO_2_ annealed at 600 °C.

Figure 10 shows the humidity dependence of the sensing response to 1 ppm acetone at an operating temperature of 180 °C for SnO_2_ annealed at 600 °C. Relative humidity is measured at room temperature. The responses of SnO_2_ at 180 °C to 1 ppm acetone are 3.333%, 3.4%, 3.458%, 3.606%, and 3.767 in an air environment with the relative humidity of 27% RH, 40% RH, 52% RH, 63% RH, and 72% RH. We can see that as the humidity increases from 27 to 72% RH, the sensing response of the SnO_2_ sensor to 1 ppm acetone increases from 3.333 to 3.487. The humidity of the environment promotes the SnO_2_ response to acetone, which is similar to LaNi_0.5_Ti_0.5_O_3_ [51] sensing properties to acetone in a humid environment. However, by comparison, the magnitude of responses in wet air (~27% RH) and wet air (~72% RH) are at the same level. The small change in response value (0.154) demonstrates that the tested environmental humidity has little effect on the SnO_2_ sensing properties of acetone vapor. This performance of SnO_2_ is different from the acetone response of La-doped α-Fe_2_O_3_ [52] and Si-doped WO_3_ [53], which decreased with increasing relative humidity.

It is well known that theoretical studies are used as a complementary tool to understand molecular reactions at an atomic level. Many DFT investigations on the sensing performance of SnO_2_ have been performed. To describe the sensitization mechanism of acetone and the SnO_2_ surface with greater clarity, we performed DFT calculations. All calculations were carried out by DFT using the program package DMol3 [54,55]. Calculations were performed with a generalized gradient approximation through the Perdew–Burke–Ernzerhof method to describe the exchange and correlation energy. Double numerical basis sets with polarization functions were adopted. The convergence criteria of optimal geometry for energy, force, and displacement were 2 × 10^−5^ Ha, 4 × 10^−3^ Ha/Å, and 5 × 10^−3^ Å, respectively. The Brillouin zone was sampled using a 3 × 3 × 1 Monkhorst Pack grid, which generated converged results for all properties. Charge transfer was calculated based on a Mulliken population analysis [56].

**Figure 11:**
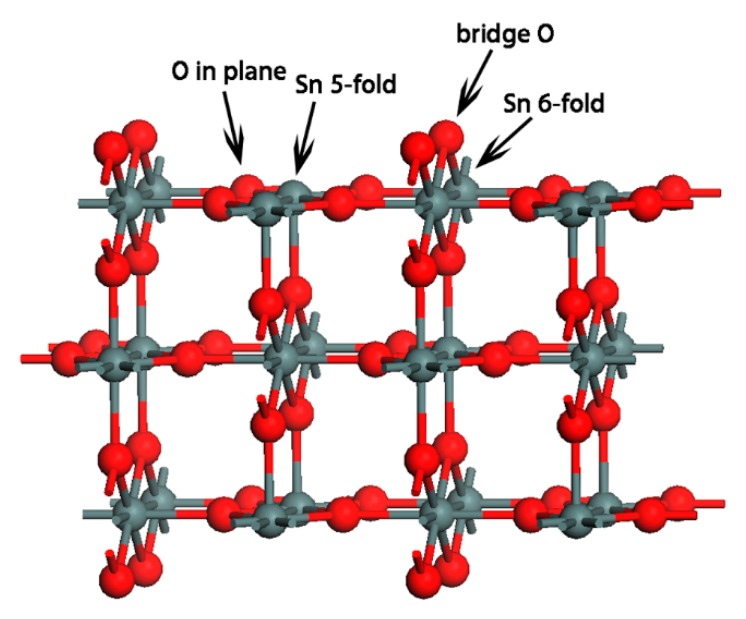
The optimized SnO_2_ (110) surface.

SnO_2_ crystal with a tetragonal structure (Pmnm (41)) was calculated with 48 atoms in the supercell. Lattice constants were *a* = 4.73735 Å, *b* = 4.73735 Å, and *c* = 3.18640 Å, and atomic fractional coordinates were Sn (0.0000, 0.0000, 0.0000), and O (0.30562, 0.30562, 0). The (110) surface was cleaved from the optimized SnO_2_ bulk, and a 10 Å vacuum was added to the layers. We chose the (110) surface of SnO_2_ because this was the most thermodynamically stable surface and has been extensively studied both experimentally and theoretically [27,30,31,33,34,35,54,55]. The optimized SnO_2_ (110) surface is shown in Figure 11. CO/H_2_-sensing behaviors and mechanisms have been experimentally found to strongly depend on the oxygen amount in the ambient, and the surface configuration of SnO_2_ reportedly depends strongly on oxygen concentration [57,58]. In the following, we investigate the adsorption of acetone for the stoichiometric, the defective and the oxygen species adsorbed SnO_2_ surface.

**Figure 12:**
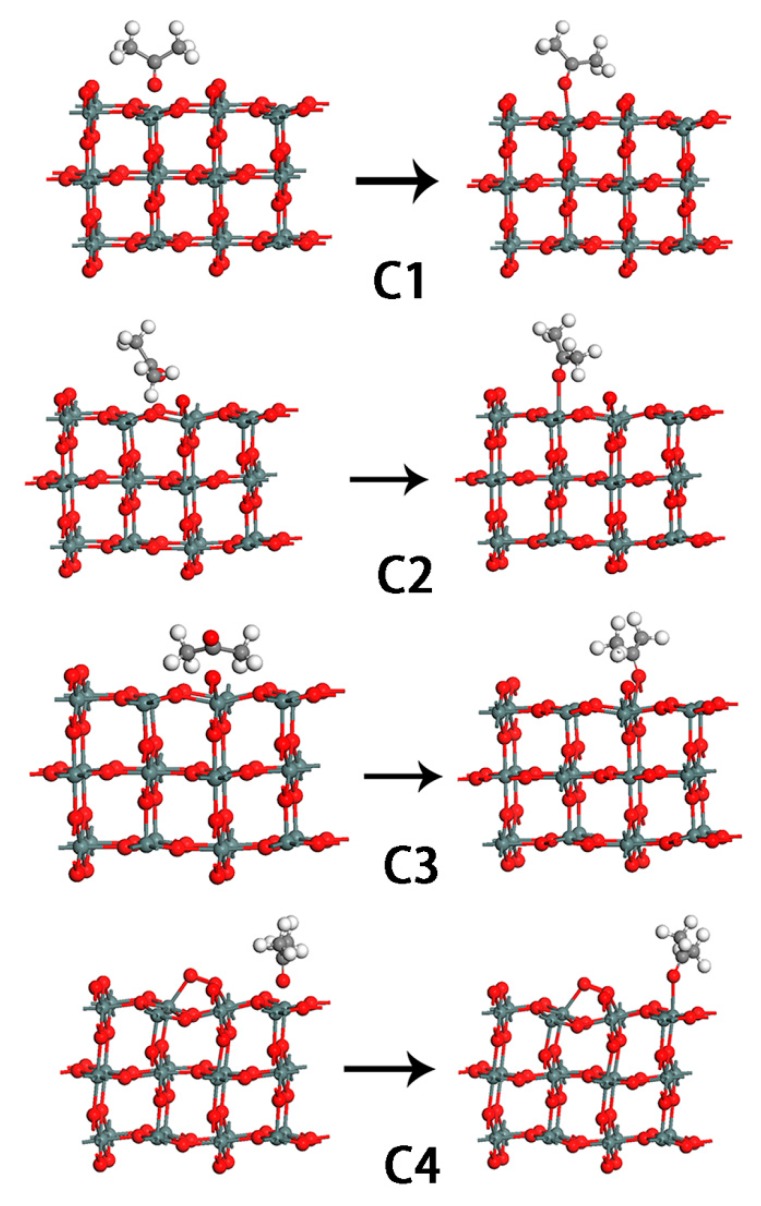
The four different adsorption configurations of acetone molecule on 2 × 2 SnO_2_ (110) surface. C1: on stoichiometric SnO_2_ surface; C2, C3: on defective SnO_2_ surface; C4: on O_2_ pre-adsorbed SnO_2_ surface.

The sign of adsorption energy can be used to clarify the possibility of the adsorption mode. Adsorption energy can be expressed as follows:

(2) $$E_{ads}=E_{substrate}+E_{adsorbate}-E_{substrate-adsorbate}$$

where $E_{substrate}$ and $E_{adsorbate}$ is the total energy of the adsorbate-substrate system in the equilibrium state; $E_{substrate}$ and $E_{adsorbate}$ are the total energy of substrate and adsorbate, respectively. In general, positive E_ads_ indicates that the interaction between the molecule and SnO_2_ surface is an exothermic process. We first chose the SnO_2_ (110) stoichiometric surface to study the reaction between acetone molecule and SnO_2_ (110). The optimized geometry is shown in Figure 11. For the stoichometric SnO_2_ surface, the acetone molecule can only be adsorbed on the five-fold-coordinated Sn, as shown in mode C1, see Figure 12. In other modes, the calculated results showed that the acetone molecule is located remotely from the SnO_2_ (110) surface after optimization. This implied that the surface sites except the five-fold-coordinated Sn of the SnO_2_ (110) stoichiometric surface were inadaptable for the acetone molecule to be absorbed. From Table 3, we could see that the optimized Sn-O_acetone_ bond length in the adsorption state was 2.23 Å, which is longer than the Sn-O bond length of SnO_2_ which is 2.11 Å. The adsorbed energy is 1.61 eV, and there are charges of 0.21 e transferred from the acetone molecule to the SnO_2_ surface during the adsorption process. In this mode, the acetone molecule acts as a donor, resulting in an increased electron concentration and reduced electrical resistance. This is consistent with previous experimental reports on n-type metal oxide semiconductors.

**Figure 13:**
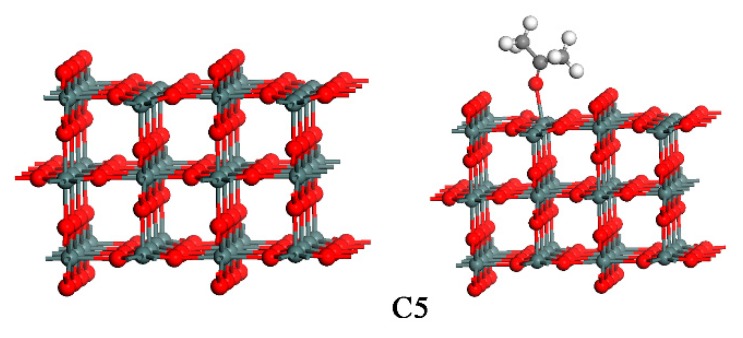
The optimized geometry of a 2 × 4 SnO_2_ (110) stoichiometric surface and the adsorption configuration of the acetone molecule on the surface.

We then chose a 2 × 4 SnO_2_ (110) stoichiometric surface to study the reaction between the acetone molecule and SnO_2_ (110). The optimized geometries are shown in Figure 13. From the calculation results, we can see that the acetone molecule can be adsorbed on a five-fold-coordinated Sn through the O atom. The geometry is similar to acetone which is adsorbed on a 2 × 2 SnO_2_ (110) stoichiometric surface. As shown in Table 3, the optimized bond length of O-Sn in equilibrium is 2.22 Å. From Table 3, we also can see that the acetone molecule acts as a donor when it is adsorbed on the Sn site on the SnO_2_ surface. In the adsorption process, the number of transferred electrons is 0.24 e. In addition, the adsorption energy *E_ads_* is 1.63 eV, which is located in the energy range of chemisorptions. The calculated results mentioned above are similar to that of acetone adsorbed on the SnO_2_ (110) stoichiometric surface, shown in mode C1. Therefore, the supercell of the surface with three layers is sufficient for acetone. Thus, we chose a 2 × 2 SnO_2_ (110) surface as the sample investigated in the present paper since the structure of this composition can be easily modeled using our calculating computer system.

In the following, we investigate the interaction between acetone molecule and SnO_2_ (110) surface with an oxygen vacancy. The bridge-bonded oxygen vacancy was created by removing one bridge O (O_b_) from the corresponding stoichiometric surface. The adsorption of acetone on the defective surface resulted in two stable geometries (mode C2 and C3), as shown in Figure 12. The bond lengths in the optimized configurations, the calculated adsorption energies, and the charge transfer are listed in Table 3. In mode C2, see Figure 12, the molecularly-adsorbed acetone binds to the surface at the five-coordinated Sn site, via the C-down mode. The adsorption energy for the molecule at this site is 1.47 eV. This value demonstrates that this is a chemical adsorption process. In mode C3, see Figure 12, the O of acetone occupies the oxygen vacancy site, bonding with the two six-coordinated Sn atoms. In this structure, the adsorption energy is 1.64 eV. The two Sn-O_acetone_ distances are 2.45 Å and 2.41 Å, which are longer than the corresponding Sn-O_b_ (bridge O) bond distance of 2.03 Å in SnO_2_ because of the decreasing covalent bond interaction between Sn and O_acetone_ atoms. Charges of 0.18 e and 0.17 e transferring from the acetone to SnO_2_ surface, respectively.

Experimental results suggest that there are adsorbed oxygen species, O^2−^ or O^−^, on the SnO_2_ surface. In order to understand the effect of adsorbed oxygen species on SnO_2_ for acetone gas sensing, we investigated the possible interaction modes between acetone and the SnO_2_ (110) surface with pre-adsorbed oxygen species O^2−^ and O^−^. For the O^2−^ adsorbed surface, the acetone can be only adsorbed on the five-fold-coordinated Sn, with the O-down mode as shown in mode C4, see Figure 12. This implies that the surface Sn site on the O^2−^ pre-adsorbed SnO_2_ (110) surface is the only adaptable site for the acetone molecule to be absorbed. The bond length of Sn-O_acetone_ is 2.31 Å. The bond length was slightly bigger than that of SnO_2_ (2.07 Å), which demonstrates that the bond interaction is becoming weaker. The adsorption energy is 1.61 eV. During the interaction process, there are charges of 0.21 e transferring from acetone to the O^2−^ adsorbed SnO_2_ surface. Simulation results showed that the acetone molecule kept far away from the O^−^ pre-adsorption SnO_2_ (110) surface after optimization, which demonstrates that the O^-^ pre-adsorption SnO_2_ (110) surface was inadaptable for the acetone molecule to be absorbed.

**Figure 14:**
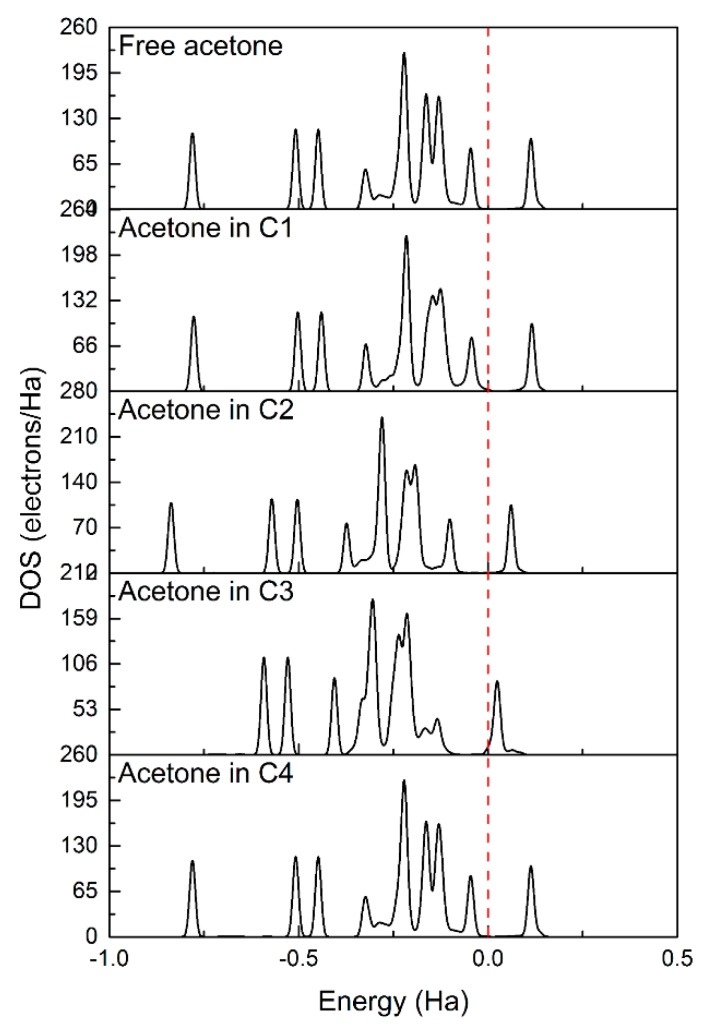
Total density of states (DOSs) of free acetone molecule and the adsorbed acetone in C1, C2, C3, and C4 mode. The vertical dotted line indicates the Fermi energy level.

**Figure 15:**
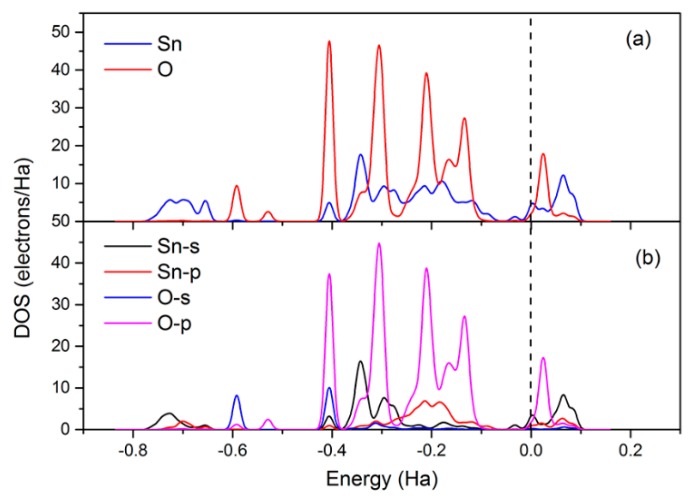
The (**a**) total density of states (DOSs) and (**b**) partial density of states (PDOSs) of O (originating from acetone) and Sn. The vertical dotted line indicates the Fermi energy level.

In order to have a clear understanding of the acetone sensing mechanism for the SnO_2_ (110) surface, we plotted the corresponding density of states (DOSs). Figure 14 showed the DOSs of a free acetone molecule and an adsorbed acetone molecule for the above modes, as shown in Figure 12. The bond energy states of adsorbed acetone molecules shifted to lower energy states compared to that of the free acetone molecules. The density of states (DOSs) of acetone molecules in C3 mode changed greatly, implying that this mode belonged to chemical adsorption. The bridging oxygen vacancy is more favorable for the adsorption of acetone than the five-fold-coordinated Sn on the SnO_2_ surface. Overall, adsorption on the vacancy is more stable than on the corresponding stoichiometric surface and the pre-adsorbed oxygen species surface. For mode C3, it could be seen from Figure 15a that there was an overlap of DOSs peaks of O (originating from acetone) and Sn. A similar phenomenon could also be seen for PDOSs peaks of O-*s*/O-*p* (originating from acetone) and Sn-*s*/Sn-*p*, as shown in Figure 15b. The results implied that there is a strong orbital hybridization between O_acetone_ orbitals and Sn orbitals.

From the above calculation results, we can conclude that on the stoichiometric surface and the O^2−^ adsorbed SnO_2_ surface, there is only one site for acetone adsorption; this is the five-fold-coordinated Sn site. On the other hand, the vacancy (V_O_) of the defective surface is also available for acetone to be adsorbed. During all the adsorption processes, electrons were transferred from acetone molecules to SnO_2_ surface, which resulted in a decrease in the resistances of the SnO_2_ sensors. Since the operating temperature has an enormous effect on the sensitivity of the sensor in real experiments, we attempt to further consider the effect of the optimal operating temperature (513 K obtained from experiments) using molecular dynamics (MD) simulation with NVT ensemble for the optimized structures, see Figure 12. The simulation results are inconsistent with the expected reactions in which the acetone will be oxidized into CO_2_ and other products. The heat-treatment applied in the simulation is perhaps inappropriate. In our following work, we will continue to investigate the gas sensing mechanism between acetone and SnO_2_.

## 4. Conclusions

In this work, we investigated the acetone sensing properties and mechanism of SnO_2_ thick-films. SnO_2_ thick film based on nanocrystalline co-precipitation powders annealed at 600 °C could sensitively detect acetone vapor. At the optimal operating temperature of 180 °C, the responses were 3.33, 3.94, 5.04, and 7.27 for SnO_2_ sensors with 1, 3, 5, and 10 ppm acetone, respectively. The density functional theory is also used to explore the acetone sensing characteristics and mechanism for n-type SnO_2_ material. DFT calculation results show that the acetone molecule can be adsorbed onto the five-fold-coordinated Sn and oxygen vacancy (V_O_) sites with O-down, accompanied by electron transfer from acetone to the SnO_2_ (110) surface. The acetone molecule acts as a donor in these modes, which is consistent with the experimental results. This could explain the reason why the resistance of SnO_2_ or n-type metal oxide decreased after the acetone molecule was introduced into the system. Molecular dynamic calculations showed that the acetone didn’t dissociate into other products such as CO_2_.

## Figures and Tables

**Table 1:** Maximum acetone sensing response and optimum operating temperature of SnO_2_ sensors.

**Annealing Temperature (°C)**	400	500	600	700	800
**Maximum Response**	1.073	1.035	1.582	1.477	1.358
**Optimum Operating Temperature (°C)**	220	220	180	180	180

**Table 2:** Acetone sensing properties for various SnO_2_ semiconductor sensors.

Materials	Preparation Method	Response	Concentration (ppm)	T_O_ (°C)	Response Time/Recovery Time (s)	Ref
Y-doped SnO_2_ hollow nanofibers	electrospinning	12.1	50	300	-/-	[3]
SnO_2_ nanowires	hydrothermal approach	6.8	20	290	-/-	[4]
0.1 wt% Ni-SnO_2_	spin-coating technique	54.2	200	350	-/-	[14]
SnO_2_ hollow microspheres	hydrothermal method	16	50	200	-/-	[18]
SnO_2_ nanotube	hydrothermal method	6.4	20	350	10/9	[22]
SnO_2_ nanowires	hydrothermal method	5.5	20	290	7/10	[23]
Aurelia-like SnO_2_	hydrothermal method	4.7	10	240	2/23	[25]
α-Fe_2_O_3_/SnO_2_ composites	hydrothermal method	16.8	100	250	3/90	[26]
SnO_2_ thin films	dip-coating	19	8	room temperature	-/-	[28]
Co-SnO_2_ thin films	spray pyrolysis technique	20	0.1	330	-/-	[29]
SnO_2_ nanobelts	electrospinning method	6.7	5	260	-/-	[36]
Ni and Ce doped SnO_2_ thick films	co-precipitation route	7.7	100	300	-/-	[38]
PbO-doped SnO_2_ thick film	sol-gel process	13.5	3500	250	-/-	[39]
RGO doped SnO_2_ nanofibers	electrospinning	10	5	350	-/-	[40]
SnO_2_ nanomaterial	thermal synthesis	18.5	100	250	4.3/156.3	[41]
SnO_2_-ZnO hetero-nanofibers	electrospinning	85	100	300	9/7	[42]
SnO_2_-TiO_2_	sol-gel method	55	200	340	100/500	[43]
SnO_2_ thick films	co-precipitation route	5.043	5	180	70/64	Present work

**Table 3:** The calculated results of different configurations: d_O-Sn_ is the O_acetone_-Sn bond length; E_ads_ is the adsorption energy; Q_acetone_ is the net charge transfer from acetone molecule to SnO_2_ surface.

Model	d_O-Sn_ (Å)	E_ads_ (eV)	Q_acetone_ (e)
C1	2.23	1.61	0.21
C2	2.34	1.47	0.18
C3	2.45	1.64	0.17
C4	2.31	1.61	0.21
C5	2.22	1.63	0.24
